# Radiographic Imaging of Parachuting-Related Ankle Fractures: Case Series

**DOI:** 10.7759/cureus.10265

**Published:** 2020-09-05

**Authors:** Dawood Tafti, Jordan Davis, Timothy Russell

**Affiliations:** 1 Department of Radiology, San Antonio Military Medical Center, San Antonio, USA; 2 Department of Radiology, Brooke Army Medical Center, San Antonio, USA; 3 Department of Radiology, Martin Army Community Hospital, Fort Benning, USA

**Keywords:** ankle fractures, parachute, ankle brace

## Abstract

Ankle sprains and fractures represent the most common cause of parachuting-related injury sustained during landing. Various factors increase risk of injury, including increased combat loads, poor weather conditions, entanglements, and night jumps. The introduction of ankle braces has decreased the incidence of ankle injuries among parachuters. Ankle radiographs are the most frequent imaging modality acquired in the initial evaluation of ankle injuries. Providers are often unfamiliar with radiographic ankle fracture patterns. We present radiographic images of 10 patients who sustained landing-related osseous fractures during the Basic Airborne Course at Fort Benning, Georgia. Understanding the frequent radiographic fracture patterns sustained during landing can help primary care providers, orthopedists, and radiologists in the initial assessment of ankle injuries in populations with high airborne operational activity and recreational parachuting.

## Introduction

Military parachute-related injuries are a relatively common occurrence, with a range of 0.9 to 29 injuries per 1000 jumps depending on various factors and weather conditions [1-4]. Ankle injuries constitute the most common injuries sustained among parachutists [1]. When considering all ankle injuries, ankle sprains are more common than the relatively infrequent ankle fractures. The Basic Airborne Course (BAC) conducted at Fort Benning, Georgia is a three-week course that requires a total of five successful jumps for graduation. Since the institution of the parachute landing fall (PLF) technique in the 1940s, military personnel around the world, including those at the BAC, are taught this technique for safe landing. The PLF technique primarily prevents ankle injuries by distributing the hard force of impact through rolling [5]. The parachutist is instructed to land with the joints in the lower extremity in slight flexion and with the feet and knees together. Upon ground impact, the parachutist allows his or her body to buckle and fall horizontally. A multi-point roll is then employed to distribute the force of impact to different parts of the body. This remains one of the most effective methods in decreasing parachute-related injury rates, considering 84% of parachute-related injuries are directly related to landing [6].

The use of the parachute ankle brace (PAB) has been shown to significantly decrease the rate of ankle sprains [4]. The use of the PAB has also been shown to decrease the rates of ankle fractures [5]. Despite these findings, PAB deployment amongst paratroopers has been inconsistent for various reasons, including discomfort and a perception that braces increase the risk of other injuries [7-9]. The patients included in our case series employed the use of braces as well as the PLF technique during their jumps. Imaging studies for ankle injuries sustained during landing frequently show nonspecific soft tissue swelling, reflecting the prevalence of ankle sprains and other soft-tissue injuries in this population. Characterization of the less-frequent parachute-related ankle fracture has been explored in the literature, albeit to a lesser degree [10]. We present 10 cases of parachute-related ankle fractures sustained by BAC trainees in order to demonstrate the different presentations of this unique traumatic entity.

## Case presentation

Case study 1

**Figure 1:**
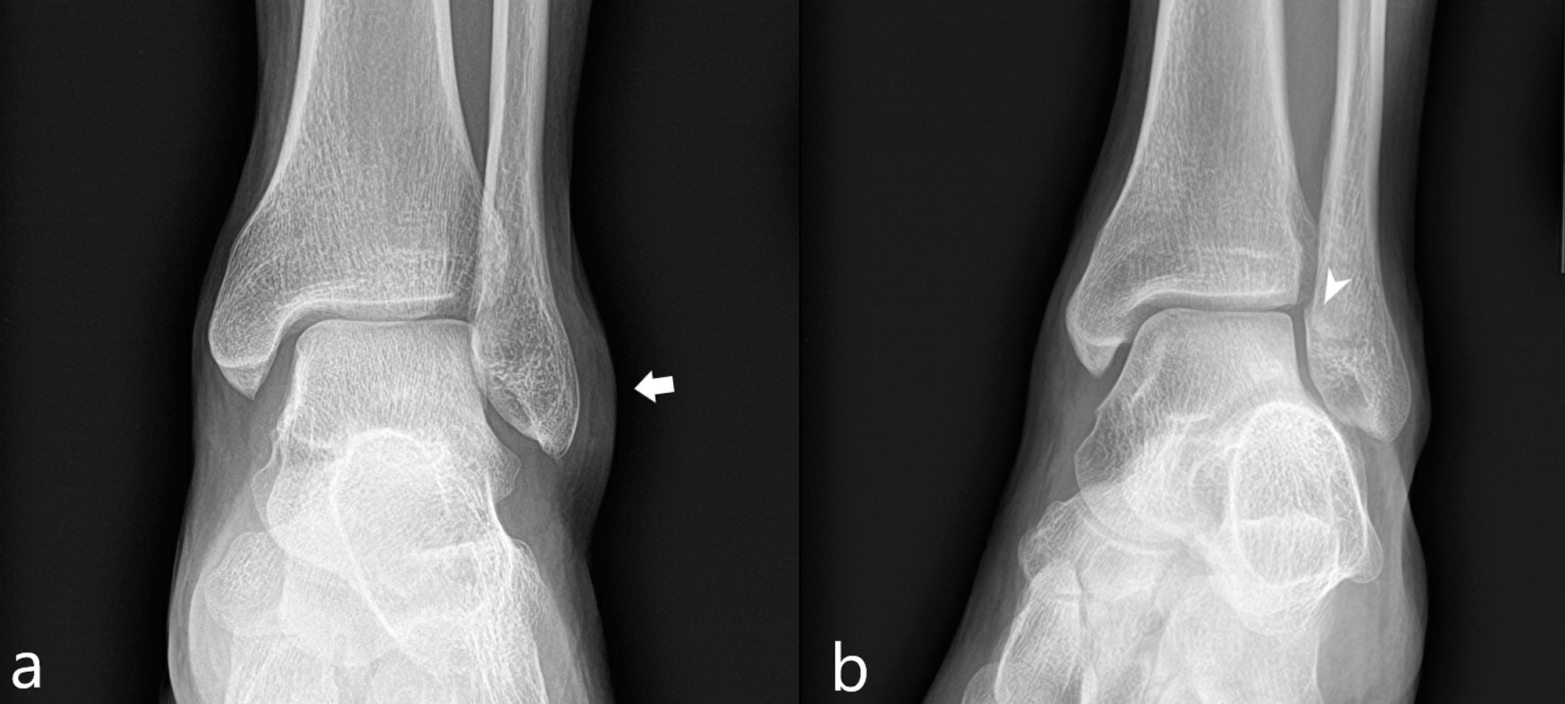
Case 1 ankle radiographs A frontal radiograph of the ankle (a) demonstrates nonspecific soft tissue swelling overlying the lateral malleolus (white arrow). An oblique mortise view radiograph of the ankle (b) demonstrates a subtle cortical lucency along the medial aspect of the lateral malleolus (white arrowhead), indicating a nondisplaced fracture.

A 27-year-old man sustained a left lateral malleolar fracture on his fifth jump. Patient’s height was 165 centimeters and his weight was 76 kilograms. Patient was treated with splinting and activity modification. Radiographs demonstrated nonspecific soft tissue swelling overlying the lateral malleolus and a subtle cortical lucency along the medial aspect of the lateral malleolus suspicious for a nondisplaced fracture (Figure 1). 

Case study 2

**Figure 2:**
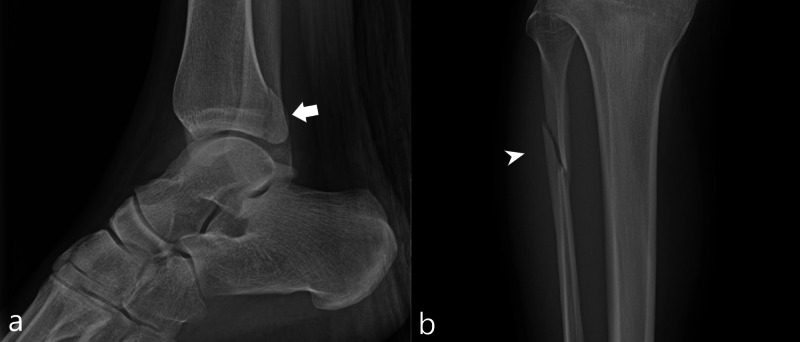
Case 2 ankle and tibia/fibula radiographs Figure 2: A lateral radiograph of the ankle (a) demonstrates a nondisplaced posterior malleolar fracture (white arrow). A frontal radiograph of the tibia (b) demonstrates an oblique fracture of the proximal fibular diaphysis (white arrowhead).

A 23-year-old man sustained right posterior malleolar and proximal fibular fractures on his third jump. Patient’s height was 196 centimeters and his weight was 95 kilograms. Radiographs of the ankle demonstrated a nondisplaced posterior malleolar fracture and an oblique fracture of the proximal fibular diaphysis (Figure 2). The patient was treated surgically with open reduction/internal fixation of the posterior malleolus.

Case study 3

**Figure 3:**
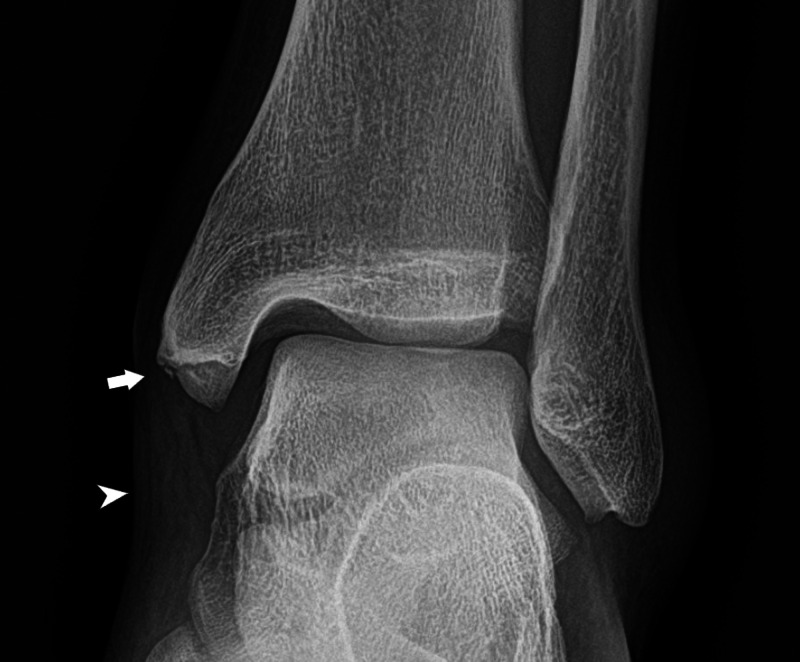
Case 3 ankle radiograph A frontal radiograph of the ankle demonstrates a small avulsion fracture of the medial malleolus (white arrow) with moderate soft tissue swelling (white arrowhead).

A 28-year-old man sustained a left medial malleolar fracture on his fifth jump. Patient’s height was 196 centimeters and his weight was 81 kilograms. According to the patient’s history, the landing was complicated by forced plantar flexion of the foot. Radiographs of the ankle demonstrated a small avulsion fracture of the medial malleolus with moderate soft tissue swelling (Figure 3). Patient was treated conservatively. 

Case study 4

**Figure 4:**
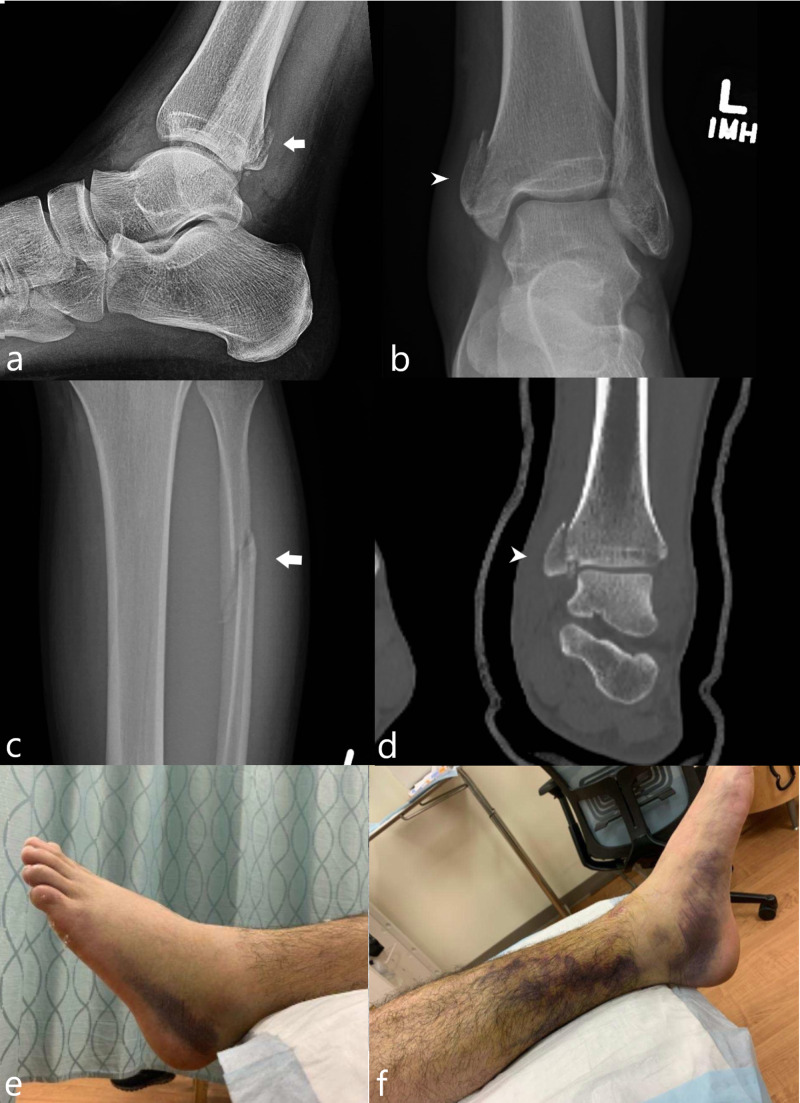
Case 4 radiologic and medical photos A lateral radiograph of the ankle (a) demonstrates a mildly displaced posterior malleolar fracture (white arrow). A frontal radiograph (b) demonstrates a mildly displaced fracture of the medial malleolus (white arrowhead).  A frontal radiograph of the proximal tibia (c) demonstrates an oblique fracture of the proximal fibular diaphysis (white arrow). A coronal computed tomography (CT) image (d) demonstrates mild comminution of the medial malleolar fracture (white arrowhead). Pictures taken after the patient sustained the landing injury (e, f) demonstrate swelling and ecchymosis.

A 29-year-old man sustained left medial malleolar and posterior malleolar fractures during his third jump. Patient’s height was 180 centimeters and his weight was 86 kilograms. According to the patient’s history, he sustained an eversion injury of the left ankle upon landing on soft mud. Radiographs demonstrated a mildly displaced posterior malleolar fracture, a mildly displaced fracture of the medial malleolus, and an oblique fracture of the proximal fibular diaphysis (Figure 4). He was treated with open reduction/internal fixation of the medial malleolus. 

Case study 5

**Figure 5:**
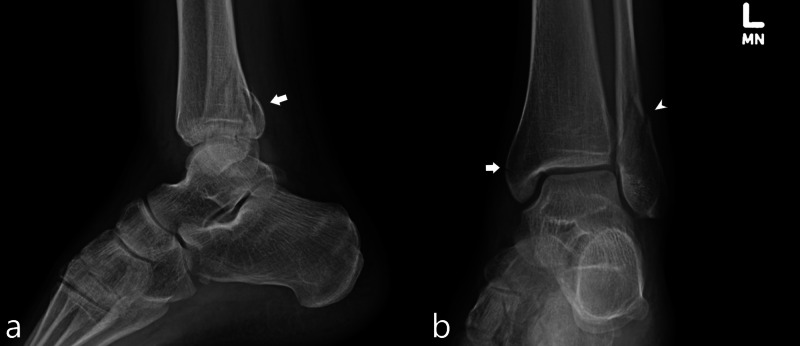
Case 5 ankle radiographs A lateral radiograph of the ankle (a) demonstrates a mildly displaced posterior malleolar fracture (white arrow). An oblique mortise view radiograph of the ankle (b) demonstrates a nondisplaced fracture of the medial malleolus (white arrow) and the lateral malleolus (white arrowhead).

A 30-year-old man sustained a trimalleolar ankle fracture during his second jump. Patient’s height was 183 centimeters and his weight was 82 kilograms. Radiographs demonstrated a mildly displaced posterior malleolar fracture and nondisplaced fractures of the medial and lateral malleoli (Figure 5).

Case study 6

**Figure 6:**
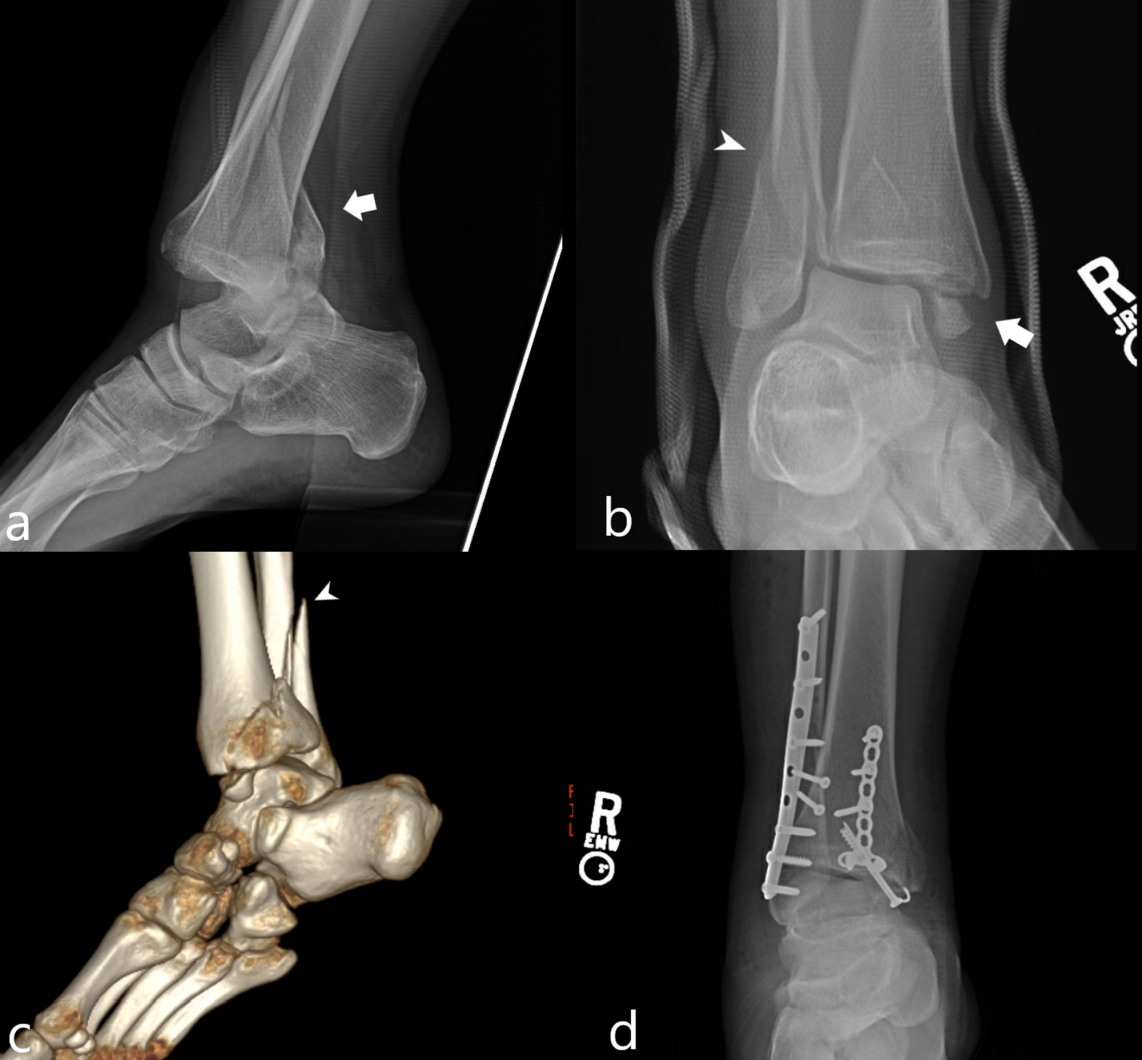
Case 6 radiologic images A lateral radiograph of the ankle (a) demonstrates tibiotalar joint dislocation as well as a fracture of the posterior malleolus (white arrow). A frontal radiograph of the ankle (b) demonstrates a nondisplaced fracture of the lateral malleolus (white arrowhead) and a moderately displaced fracture of the medial malleolus (white arrow). A reconstructed 3D image (c) demonstrates the lateral malleolar fracture to a better degree. A post-operative frontal radiograph (d) demonstrates open reduction/internal fixation of the trimalleolar ankle fracture.

A 23-year-old man sustained a right trimalleolar ankle fracture during his first jump. Patient’s height was 188 centimeters and his weight was 104 kilograms. Radiographs demonstrated a tibiotalar joint dislocation as well as a fractures of the posterior, lateral, and medial malleoli (Figure 6). He underwent open reduction/internal fixation.

Case study 7

**Figure 7:**
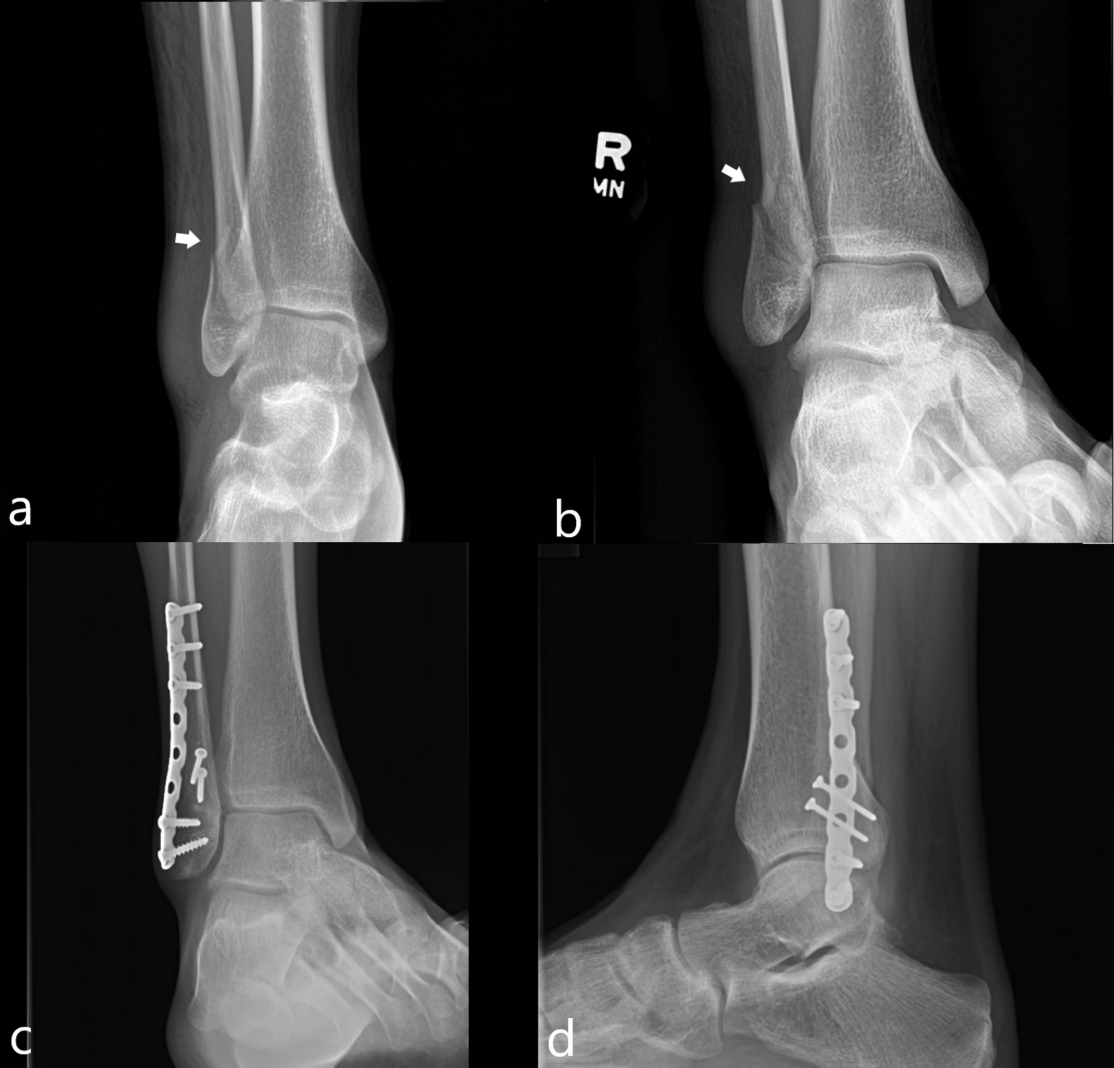
Case 7 radiographic images of the ankle A frontal radiograph of the ankle (a) demonstrates a mildly comminuted fracture of the lateral malleolus(white arrow). An oblique mortise view radiograph of the ankle (b) demonstrates the fracture to a better extent (white arrow). Oblique mortise view (c) and lateral (d) radiographs demonstrate changes related to internal fixation.

A 25-year-old man sustained a right lateral malleolar fracture during his third jump. Patient’s height was 188 centimeters and his weight was 81 kilograms. Radiographs of the ankle demonstrated a mildly comminuted fracture of the lateral malleolus (Figure 7).

Case study 8

**Figure 8:**
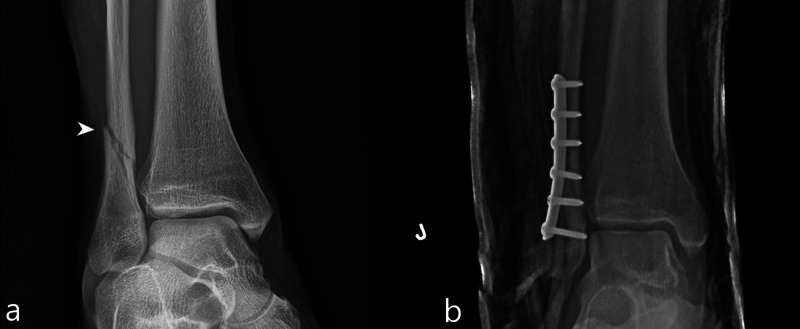
Case 8 radiographic images of the ankle A frontal radiograph of the ankle demonstrates a non-comminuted fracture of the lateral malleolus (white arrowhead). A frontal radiograph of the ankle (b) demonstrates changes related to internal fixation.

A 28-year-old man sustained a right lateral malleolar fracture during his fifth jump. Patient’s height was 183 centimeters and his weight was 77 kilograms. Radiographs of the ankle demonstrated a non-comminuted fracture of the lateral malleolus (Figure 8).

Case study 9

**Figure 9:**
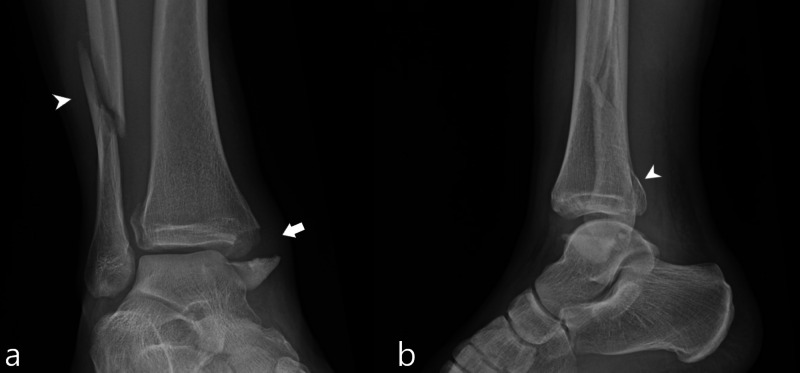
Case 9 radiographic images of the ankle A frontal radiograph of the ankle (a) demonstrates a nondisplaced fracture of the lateral malleolus (white arrowhead) and a moderately displaced fracture of the medial malleolus (white arrow). A lateral radiograph of the ankle (b) demonstrates a mildly displaced posterior malleolar fracture (white arrowhead).

A 23-year-old male sustained a right trimalleolar ankle fracture during his third jump. Patient’s height was 180 centimeters and his weight was 76 kilograms. Radiographs demonstrated a nondisplaced fracture of the lateral malleolus, a moderately displaced fracture of the medial malleolus, and a mildly displaced posterior malleolar fracture (Figure 9). He was treated with open reduction/internal fixation.

Case study 10

**Figure 10:**
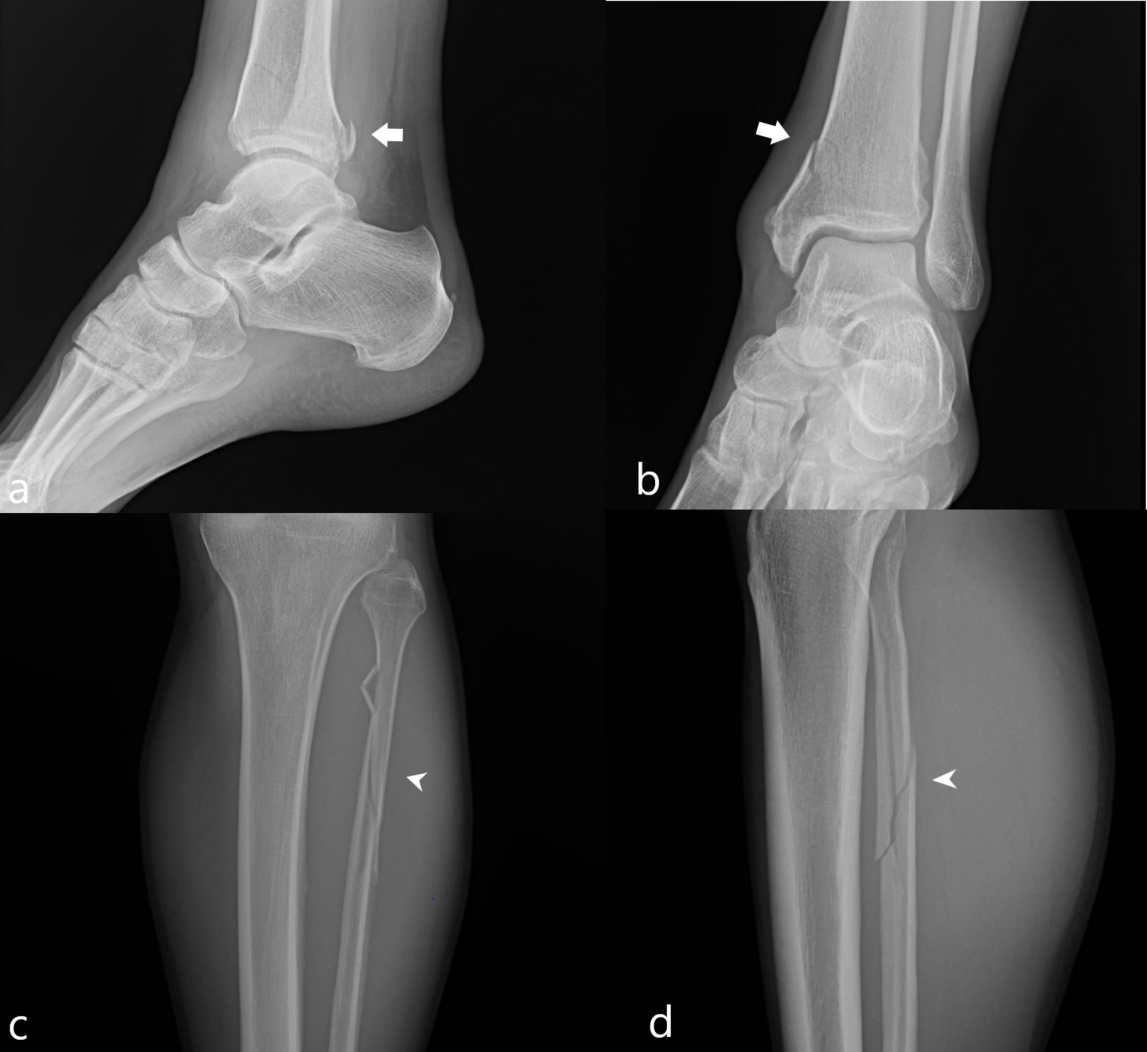
Case 10 radiographic images of the ankle and tibia/fibula A lateral radiograph of the ankle (a) demonstrates a mildly displaced fracture of the posterior malleolus (white arrow). An oblique mortise view radiograph of the ankle (b) demonstrates a nondisplaced fracture of the medial malleolus (white arrow). Frontal (c) and lateral (d) radiographs of the tibia demonstrate a spiral fracture of the proximal fibula (white arrowhead). The constellation of findings is consistent with a Maisonneuve fracture.

A 42-year-old male sustained left medial malleolar, proximal fibular diaphyseal, and posterior malleolar fractures during his first jump. Patient’s height was 180 centimeters and his weight of 86 kilograms. Radiographs demonstrated a mildly displaced fracture of the posterior malleolus, a nondisplaced fracture of the medial malleolus, and a spiral fracture of the proximal fibula consistent with a Maisonneuve fracture (Figure 10).

Summary of the demographic data is shown below (Table 1). The patients' ages ranged from 23 to 42 years. All patients were male. Body mass index (BMI), site of injury, fracture site(s) and jump numbers are also provided.

**Table 1:** Table summary of cases Summary of patient information in this case series. LM = lateral malleolus; PM = posterior malleolus; MM = medial malleolus; F = fibula.

Case study	Age	Side of injury	Sex	BMI (kg/m2)	Fracture Site	Jump number
1	27	Left	Male	26.62	LM	5
2	23	Right	Male	24.9	PM, F	3
3	28	Left	Male	20.99	MM	5
4	29	Left	Male	26.51	MM, PM, F	3
5	30	Left	Male	24.41	MM, PM, LM	2
6	23	Right	Male	29.53	LM	1
7	25	Right	Male	22.96	LM	3
8	28	Right	Male	23.08	LM	5
9	23	Right	Male	23.43	LM, MM, PM	3
10	42	Left	Male	26.4	MM, PM, F	1

**Figure 11:**
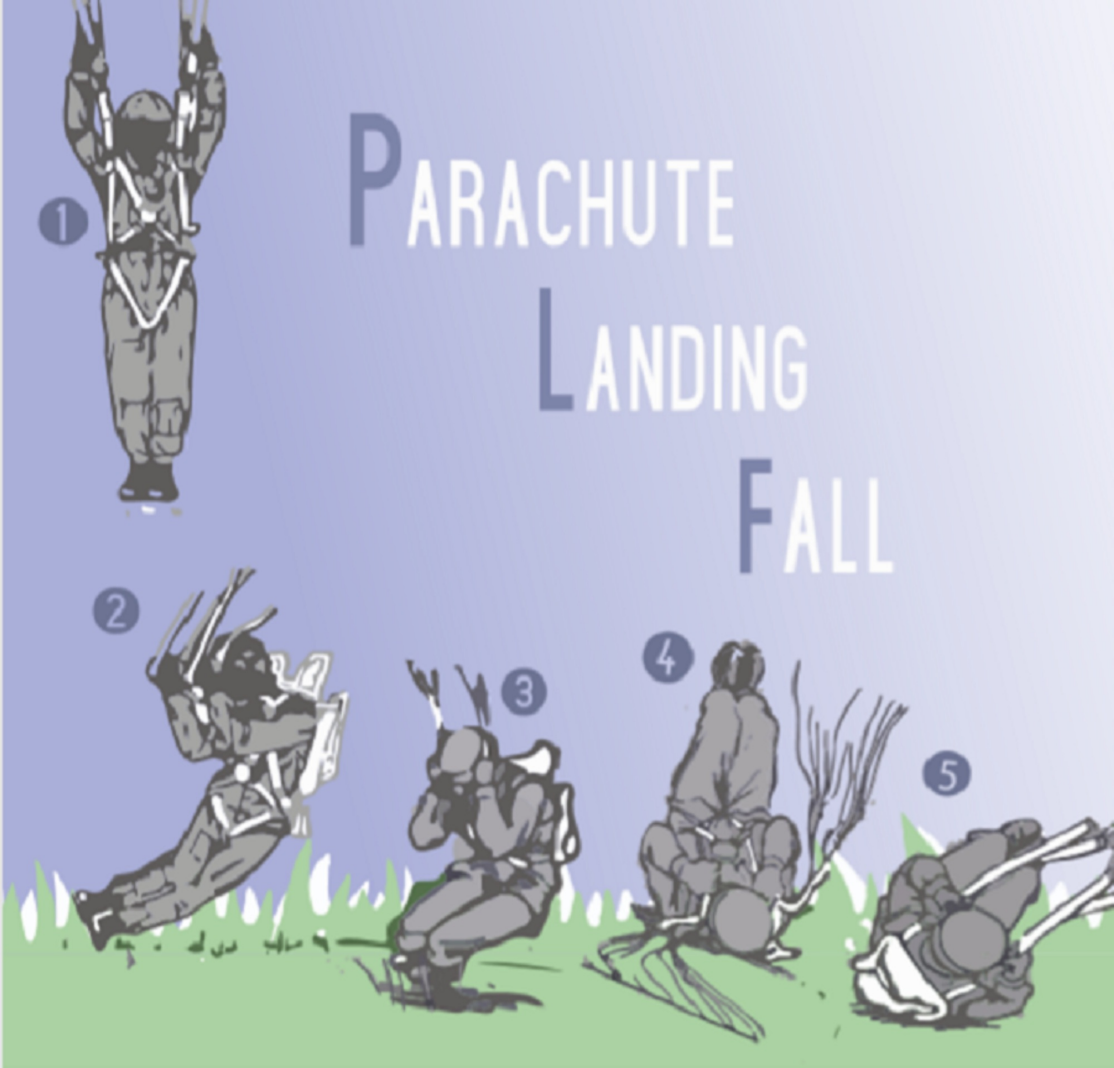
Illustration of the parachute landing fall An illustration of the parachute landing fall (PLF). (1) The parachutist initially descends in the vertical position in the direction of travel with the knees and feet together. (2) Moments before impact, the parachutist slightly flexes the knees in preparation of assuming a horizontal position. (3) The parachutist allows his body to buckle and proceeds to absorb the energy of impact by going towards the horizontal position. (4 and 5) The parachutist continues to roll in a horizontal position, distributing the energy of impact throughout his/her body. Illustration courtesy of Alana Drummond.

The PLF technique distributes the hard force of impact through rolling (Figure 11) The parachutist is instructed to land with the joints in the lower extremity in slight flexion and with the feet and knees together, while then allowing the body to buckle and fall horizontally upon ground impact. A multi-point roll is then employed and distributes the force of impact to different parts of the body [11]. 

## Discussion

Various landing conditions predispose to injury, including landing on dirt strips, night operations, and poor weather conditions [2,12]. Certain traumatic landing mechanisms cause classic fracture configurations of the ankle. For example, landing in plantar flexion (toes pointing down) distributes the force of ground impact to the metatarsals as well the tibiotalar joint, leading to a possible posterior malleolar fracture [1]. While attempting the PLF, the ankle striking the ground is first subjected inversion, possibly leading to a fracture of the lateral malleolus.

The ankle which does not immediately strike the ground can also be subjected to eversion, possibly leading to a medial malleolar fracture. Fractures of the upper third of the fibula, as demonstrated in three of our patients, are also a characteristic fracture sustained during landing injuries. Other mechanisms predisposing to injury include double-leg landing, which distributes force to the knees and femurs, and single-leg landing, which distributes force to the ankle and hip [13]. In one retrospective study, the posterior malleolar fracture has been shown to constitute 41% of fractures sustained in parachute landings [10]. 

Our trainee population is unique in certain respects when compared to other populations. Although inexperience is a risk factor for parachute-related injury, trainees sustain injuries less frequently than operational personnel [2]. There is also a more heterogeneous distribution of injuries among operational personnel when compared to trainees [2]. Our trainee population employs the use of PAB, which is often not used by operational personnel. Use of the PAB likely prevents medial and lateral malleolar fractures due to prevention of hypereversion and hyperinversion. The role of PAB in the prevention of posterior malleolar fractures is unclear, however it is likely less effective in preventing forced plantar flexion injuries.

## Conclusions

Ankle fractures are among the most common fractures to be treated by orthopedic surgeons. Ankle injuries are also the most common presentation of landing-related injuries in parachutists. Our case series demonstrated a wide range of fracture morphologies in order to better illustrate the varied mechanisms of injuries sustained among trainees. Providers taking care of a military population with airborne activity should familiarize themselves with the frequent radiologic patterns of ankle fractures sustained during landing.
